# Supplementary material for: Chromothripsis during telomere crisis is independent of NHEJ, and consistent with a replicative origin
Source: Genome Res. 2019 May;29(5):737–49. doi: 10.1101/gr.240705.118 (PMC6499312; doi:10.1101/gr.240705.118)
Supplement: Supplemental Material [file supp_gr.240705.118_Supplemental_file_1.zip › contigs/annotated_contigs/DB73/contig.3.DB73_length_605_mean_cov_12.5752066116.docx]

**DB73_length_605_mean_cov_12.5752066116**

```
AATTTCCAGCCCTGTAATCTTTTCACTGTAAAACATTCCTGTACCTCTTCTCTTTAAGATGTTATTGGTGGGGGACAGGGTTGGTGGGC
 >chr3:189559842-189560159 - E=4e-179
AGATCGGAGAGCTTTATGGACACAGACGTGACATACACGCGCTCTCTCTCTCTCTCTCTCCAGACAGAAGACAATACGTTGCAACCCCC

TTCCACTGCAGTAGCAGCATGTCTGTCTCAGCACACTGCTCTGCAAGTCAGCTATCATTTTCCCTTAAGGTAGAGAAAACATTATCCTC

CCCCTAAGGGTGGCTTGCTACTCCCAACTGCCTGCAAAACCGCGGGCTAC|AAAACTGTTAATTACTTGTTAATTGAATCAAATTTATC

ATTTGATACAGTGTAT|CAAGACAAAGAACCATGCCCTATTGAAGCTTTTGT|GCTT|CTATTGAAGCTTTTGTTCAGCCCTCAAATCA
                 >chr3:188573476-188573511 + E=2e-09 p=0e+00  >chr3:188573495-18857369
CTCCATTTCAAGGGACCAAAAGCCCTATTAACTTAATTCCCTTATTTTATAGCTGACGGAATTTAGGCCCAGGGAGGAAATGCTTTGTA
0 + E=1e-105
CAAAACTCACTGCTGGTGGCTGACGTTGGCAAGACTGGAATACAGCCCTTGACTTCTGCTTTATTTCTCCTTGCC
```
